# “History, Theory and Ethics of Medicine”: The Last Ten Years. A Survey of Course Content, Methods and Structural Preconditions at Twenty-nine German Medical Faculties

**DOI:** 10.3205/zma001100

**Published:** 2017-05-15

**Authors:** Jan Schildmann, Florian Bruns, Volker Hess, Jochen Vollmann

**Affiliations:** 1 Ruhr-Universität Bochum, Institut für Medizinische Ethik und Geschichte der Medizin, Bochum, Germany; 2 Charité - Universitätsmedizin Berlin, Institut für Geschichte der Medizin und Ethik in der Medizin, Berlin Germany

**Keywords:** medical history, medical theory, medical ethics, medical studies, curricula teaching, survey

## Abstract

**Objective: **“History, Theory, Ethics of Medicine” (German: “Geschichte, Theorie, Ethik der Medizin”, abbreviation: GTE) forms part of the obligatory curriculum for medical students in Germany since the winter semester 2003/2004. This paper presents the results of a national survey on the contents, methods and framework of GTE teaching.

**Methods: **Semi-structured questionnaire dispatched in July 2014 to 38 institutions responsible for GTE teaching. Descriptive analysis of quantitative data and content analysis of free-text answers.

**Results: **It was possible to collect data from 29 institutes responsible for GTE teaching (response: 76%). There is at least one professorial chair for GTE in 19 faculties; two professorial chairs or professorships remained vacant at the time of the survey. The number of students taught per academic year ranges from <100 to >350. Teaching in GTE comprises an average of 2.18 hours per week per semester (min: 1, max: 6). Teaching in GTE is proportionally distributed according to an arithmetic average as follows: history: 35.4%, theory 14.7% and ethics 49.9%. Written learning objectives were formulated for GTE in 24 faculties. The preferred themes of teaching in history, theory or ethics which according to respondents should be taught comprise a broad spectrum and vary. Teaching in ethics (79 from a max. of 81 possible points) is, when compared to history (61/81) and theory (53/81), attributed the most significance for the training of medical doctors.

**Conclusion: **10 years after the introduction of GTE the number of students and the personnel resources available at the institutions vary considerably. In light of the differences regarding the content elicited in this study the pros and cons of heterogeneity in GTE should be discussed.

## Zusammenfassung

**Zielsetzung: **Der Querschnittsbereich „Geschichte, Theorie, Ethik der Medizin (GTE)“ gehört seit dem Wintersemester 2003/2004 zum Pflichtcurriculum für Medizinstudierende. In dieser Arbeit werden die Ergebnisse einer nationalen Umfrage zu Inhalten, Methoden und Rahmenbedingungen der GTE-Lehre vorgestellt.

**Methodik: **Semistrukturierter Fragebogen, der im Juli 2014 an 38 für die Lehre in GTE verantwortliche Institutionen versandt wurde. Deskriptive Analyse quantitativer Daten und Inhaltsanalyse der Freitextantworten.

**Ergebnisse: **Es konnten Daten von 29 für die GTE-Lehre verantwortlichen Institutionen erhoben werden (Rücklauf: 76%). An 19 Fakultäten ist mindestens eine Professorenstelle für ein Arbeitsgebiet des Querschnittsbereichs besetzt; zwei Lehrstühle bzw. Professuren sind zum Zeitpunkt der Befragung unbesetzt. Die Anzahl der unterrichteten Studierenden pro Studienjahr reicht von < 100 bis > 350. Die Lehre in GTE umfasst durchschnittlich 2,18 SWS (Min: 1, Max: 6). Die SWS verteilen sich anteilig im arithmetischen Mittel wie folgt: Geschichte: 35,4%, Theorie 14,7% und Ethik 49,9%. An 24 Fakultäten wurden schriftlich Lernziele für GTE formuliert. Die von den Befragten befürworteten Themen, die in den Arbeitsgebieten Geschichte, Theorie beziehungsweise Ethik gelehrt werden sollen, umfassen ein breites Spektrum und variieren. Der Lehre in Ethik (79 von max. zu vergebenden 81 Punkten) wird im Vergleich zur Geschichte (61/81) und der Theorie (53/81) die größte Bedeutung für die Ausbildung zum Arzt zugeordnet.

**Schlussfolgerungen:** Zehn Jahre nach Einführung von GTE in das Medizinstudium variieren die Anzahl der in der Pflichtlehre zu unterrichtenden Studierenden sowie die personelle Ausstattung der Institute erheblich. Die ermittelten Unterschiede bei den Lehrinhalten sollten mit Blick auf Vor- und Nachteile der Heterogenität im Querschnittsbereich diskutiert werden.

## 1. Introduction and Problem

 “History, Theory and Ethics of Medicine” (German: “Geschichte, Theorie, Ethik der Medizin”, abbreviation: GTE) has constituted part of the compulsory curriculum of medical studies since the winter semester of 2003/2004 after the implementation of the amended regulations for licensing doctors (ÄAppO). Furthermore, the newly reformed ÄAppO rates the “intellectual, historical and ethical foundations of medical conduct” among the most important objectives in medical training [https://www.gesetze-im-internet.de/_appro_2002/BJNR240500002.html]. In order to demarcate the framework regarding the content of this newly established field of teaching and to provide those teaching staff responsible with orientation, the Akademie für Ethik in der Medizin, the Fachverband Medizingeschichte as well as other institutions elaborated teaching and learning objectives for teaching on the subjects of history, theory and medical ethics [1], [2], [3]. In addition, by way of new GTE text books, an attempt was made to familiarise students with the subjects and to facilitate the didactic integration of history, theory and ethics [4], [5], [6], [7], [8]. A 2004 special issue of the journal “Zeitschrift für medizinische Ethik” presented the structure of medical ethics teaching in selected faculties at that time; the single contributions already indicated what could be confirmed by a 2006 survey on the state of GTE in German universities which showed quite a heterogeneous teaching situation. The GTE courses differed from one another considerably in form, content and method [9]. 

In view of the three distinct working areas of history, theory and ethics which form part of GTE, it comes as no surprise that the broad thematic spectrum for those responsible for GTE continued to represent a challenge [10], [11]. This holds all the more given that the institutions responsible for teaching place very different emphases on teaching and research regarding the three different fields history, theory and ethics of medicine. Thus, ten years after the introduction of GTE, a nationwide investigation seemed of interest to identify common features and differences regarding content and methods of teaching in GTE. In this respect, we were particularly interested in the following aspects: In view of the tight time budget for the curricula, what priorities are set between history, theory and ethics in terms of content and methodology? What structural frameworks in the faculties are available to teaching ten years after the implementation of GTE? What problems have been pointed out by teaching staff regarding the implementation of teaching in GTE? The present paper summarises, for the first time, the results of the survey and discusses the further development of the cross-section area in teaching.

## 2. Methods

A semi-structured questionnaire was developed based on the authors’ content interests and a selected literature research in German-speaking periodicals. As a result, one part of the group of questions was elaborated pursuant to a national survey carried out on the subject by Möller et al. in 2006 [9]. A first draft of the initial questionnaire was followed by a pretest among six researchers teaching in GTE, which provided written and oral responses on content, comprehensibility and form. The final version of the questionnaire was composed following revisions and renewed testing by the group of authors. The questions are predominantly ordinal-scale, with predefined response possibilities. As a supplement to this form of questions, the questionnaire contains evaluative statements, which could be answered with the aid of a five-tier Likert scale (1=absolute rejection of the statement; 5=absolute agreement with the statement). The possibility of giving an answer was provided by free text-fields in the case of a few open questions. The questionnaire is available from the authors on request. 

The directors of the GTE institutes in Germany were informed about the research project within the framework of a conference held in March 2014. The questionnaire was dispatched to the heads of the 38 institutes or departments responsible for GTE in July 2014. Identification of the contact persons was carried out based on the Internet presentations of the respective faculties of medicine or universities in Germany, as well as in supplementary e-mail correspondence or telephone discussions. An electronic reminder with the questionnaire attached as a PDF file was subsequently dispatched in September 2014. The plausibility of the results and possible interpretations of the data were also discussed for the purposes of exchanges within the group of authors, along with four heads of GTE institutes as part of a 20- to 30-minute interview. The option of supplementary information from entries contained in the institutes’ websites was waived owing to differences regarding topicality and content. 

The results of the written evaluations are presented below. Analysis of anonymous data was descriptive; average values were specified as arithmetic mean.

## 3. Results

Data from 29 of those institutes responsible for GTE teaching could be collected (76% response). Nineteen faculties teach GTE as part of the regular course in medical studies and five faculties teach GTE as part of a reformed or model course of studies. A parallel model curriculum also exists alongside the regular curriculum at four faculties; one faculty could not be allocated to any of these categories.

### Organisational and Structural Frameworks

At the time the questionnaire was conducted, a professor or professorial chair for one of the three fields (history, theory, ethics of medicine) was occupied in nineteen faculties. One institute had a provisional director, while, in another, the professorial chair was unoccupied. Lectureships for GTE exist at two faculties; teachers at two further locations are attached to the Research Ethics Commission or directly to the deanship (staff position). Parts of the GTE courses at three locations are supported by other departments. In one case, no specific instructions were given. An average of 2.8 permanent posts (full-time equivalents) for teaching GTE are available per institute, whereby personnel resources are indicated between a minimum of 0 and a maximum of 9 permanent posts. The number of students taught per academic year ranges from <100 to >350. 

**Figure 1:**
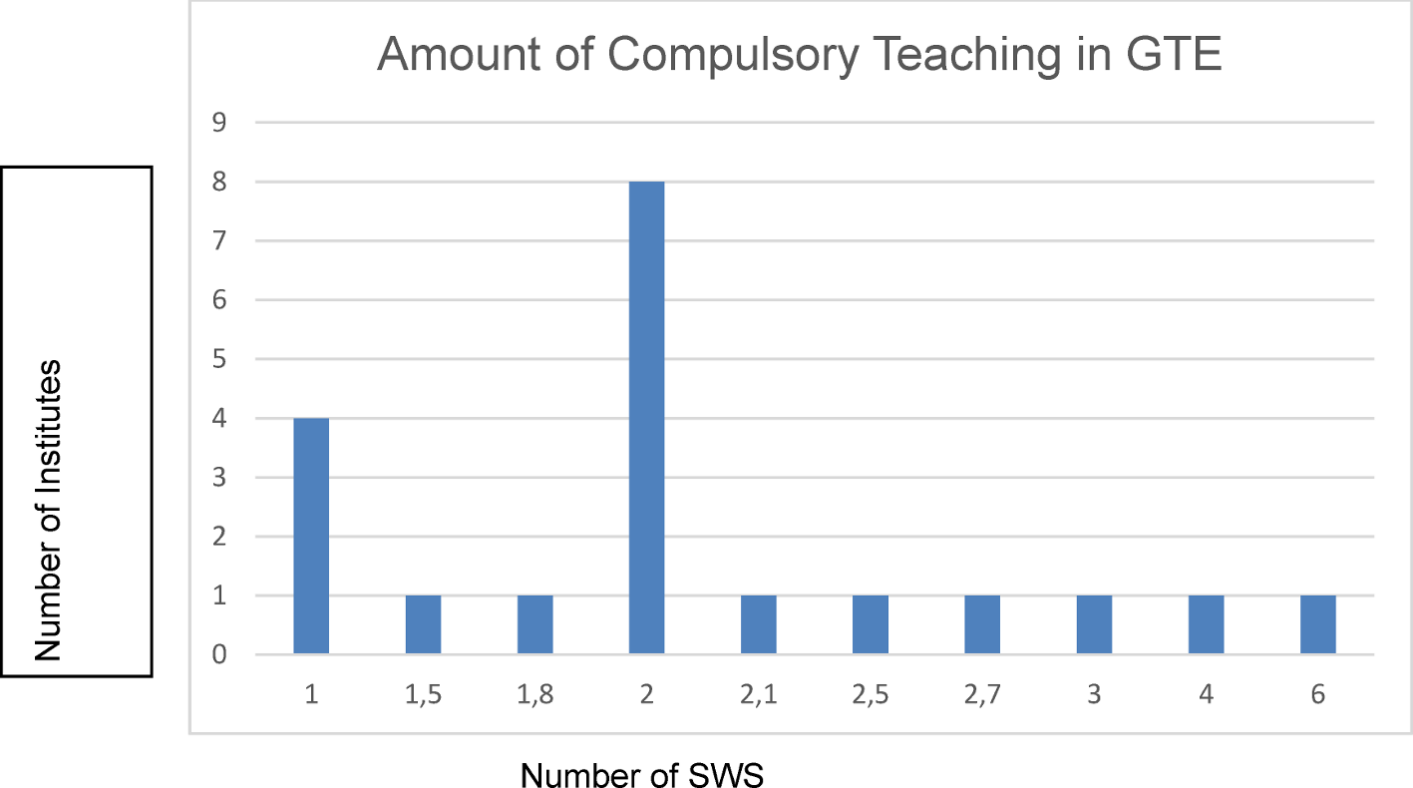
Number of compulsory courses in GTE in SWS

In four institutes the number of compulsory courses in the cross-section area was indicated by one teaching hour (45 minutes) per week per semester (German abbreviation: SWS). Six SWS were indicated as the maximum value at one institute. Based on information from 20 institutes, an average value of 2.18 SWS (arithmetic average) could be determined (see Figure 1 (Fig. 1)). The course scope at three additional faculties was not indicated in SWS, but as the total number of hours, ranging between 20 and 30 course hours. Information from six faculties was missing, or could not be evaluated.

Usable information for quantitative evaluation in each of the three fields of GTE (history, theory, ethics) could be extracted from 17 questionnaires. Accordingly, the teaching was distributed according to arithmetic averages as follows: history: 35.4%, theory 14.7% and ethics 49.9%. Two institutes made explicit reference to integrated teaching instead of itemisation. The teaching of compulsory GTE courses is, for the most part, distributed over several semesters, whereby the greater part of the courses is held in the fifth semester (N=10). One of the most frequent requirements cited regarding improved teaching in GTE was for more personnel (N=10), more SWS (N=9) and stronger accounting for GTE in the curriculum planning (N=7).

#### Methods of Teaching and Examination 

24 institutions indicated that they had written teaching objectives for GTE courses. At 20 faculties the common policy paper of the Fachverband Medizingeschichte and the Akademie für Ethik in der Medizin [2] was used as a basis for teaching. In addition to the most frequently used teaching methods, lectures (N=26) and case studies (N=21), in some faculties, source analyses (N=15) or discussions with simulated patients (N=5) were also used as teaching methods in the *compulsory courses*. Combinations of two or more examination types were predominant (N=16) whereby the respective combinations vary considerably. The most frequent combinations (in each case N=3) consist of multiple choice (MC) exams and assignments of MC tests, assignments, presentations and case analyses. The single examination method cited most frequently is the MC exam with a minimum of 50% ordinal-scale questions (N=10). A written exam with exclusively open questions was used for examination in one faculty, and in another, no separate examination in GTE took place; instead, the written modular final examination contained several MC questions on GTE. None of the participating faculties surveyed used objective structured clinical examinations (OSCE) for the examination of GTE compulsory classes.

#### Teaching Content and Co-operation with Other Subjects

The majority of institutes (N=21) favoured an “integrated teaching” of the content of history, theory and ethics in medicine. Data about agreement or rejection with regards to an integrated teaching of history, theory and ethics as part of GTE were elicited by way of a five-tier scale (see Table 1 (Tab. 1)).

#### Significance for Training 

Notwithstanding the fact that the proof of performance in GTE does not differentiate according the three subjects of history, theory and ethics of medicine, we were interested to measure the attributed significance of the three areas of history (G), theory (T) and ethics (E) for medical training. For this purpose, points could be awarded respectively for G, T and E: 3 points – very important, 2 points – important, 1 point – less important. The different categories (e.g. 3 points for very important) could also be awarded for two or all three subjects by each of the 27 institutions which completed this question. Thus, a maximum of 81 points was attainable for each area. Teaching in the area of ethics was allocated the greatest significance for medical training (79 from 81 points), followed by history (61/81) and theory (53/81). 

Respondents could provide concrete information in open, free-text answers regarding the subject fields preferred in GTE. Based on free-text answers, Table 2 (Tab. 2) summarises the topics which according to the respondents should be taught in history, theory or ethics of medicine. 

19 of the 29 institutes which provided answers indicate that compulsory GTE courses were carried out in co-operation with other subjects. Departments of anaesthesiology, intensive care, psychiatry and palliative medicine were cited as the most frequent co-operation partners in the area of GTE.

#### Framework and Problems in Teaching GTE

In the concluding questions of the survey we asked about the structural framework for GTE teaching at the respective institutions. We also asked about possible difficulties associated with teaching GTE. According to free-text comments most respondents mentioned the lack of staff positions (ten citations). Further problems cited were an insufficient number of teaching hours for teaching GTE content (nine citations) and too low an estimation of GTE on side of the faculties (seven citations) and students (six citations). Three respondents formulated as basic requirement the foundation of an institute for teaching of GTE. 

## 4. Discussion

Ten years after the introduction of GTE our data indicate that the content, the teaching methods and the structural preconditions for the teaching of GTE vary strongly. Compared to the situation in 2004 [9], there continues to be a heterogeneous range of teaching courses. This diversity appears to have been consolidated as a special feature of GTE. A professorial chair for at least one of the working fields of “History, Theory, Ethics of Medicine” does not exist in all faculties. The number of posts indicated as being available for teaching varies considerably according to location. The interpretation of the reported figures should be accounted with caution since the self-declaration of posts may be distorted due to misunderstandings of the questionnaire (e.g. regarding the full-time equivalent or inclusion of third-party funding). 

An average of 2.18 SWS are taught at the faculties, whereby the teaching of medical ethical content occupies the greatest amount of time. With 49,9% the share of the teaching of ethics as part of GTE is reported to be higher in this study compared to earlier estimates, which were assumed to be about 40% [12]. Thus – in some senses correspondingly – respondents attributed the greatest amount of significance of GTE teaching to the field of ethics for the training of medical doctors. There are also data from a survey of medical students in two faculties conducted in 2012 which indicate that on side of the students greater importance is attributed to courses in medical ethics [13]. Nevertheless, according to existing regulations on licensing doctors teaching of medical history, medical theory, and medical ethics exists without any distinction or prioritisation of the sub-subjects. Similarly, the proof of performance refers to GTE as a whole. A clear majority of respondents favoured an integrated teaching of historical, theoretical and ethical content as part of GTE. Justification for such integrated teaching could be, apart from topic specific connections between the three sub-subjects, the overarching humanities orientation in GTE with its focus on a critical reflection on natural science dominated medicine [14]. 

Among the most important topics mentioned were medicine during the National Socialist period (N=11) for history, concepts of health or illness (N=9) for theory and dilemmas at the end of life for ethics (N=17). Given that GTE already covers a complex and broad thematic area, a broad range of themes was mentioned in particular for the sub-subjects of medical ethics and medical history. According to our estimation, there are two essential reasons for these differences which should be discussed:

1. the different scientific profiles of the institutes responsible for the courses, and
2. the different curricula requirements within the respective faculties which, through the increased development of so-called “model” or “reformed” curricula of medical studies, continue to become increasingly more differentiated.

The emergence of the “model curricula” has led at some faculties to the fact that GTE is no longer available as a defined, more or less clearly outlined teaching area. Instead the corresponding topics and methods are to be found, if at all, distributed over single modules. In this respect, one must critically consider whether the foundations of humanities as overarching feature of GTE can be adequately taught in such curricula. At the same time, those responsible for teaching in GTE must ask themselves whether, in the little time available for teaching and considering the numerous possible topics, each of which must be processed (e.g. focus on theoretically relevant aspects or on questions arising on a daily basis in the clinical context). Standardisation of teaching should be advocated in the sense of a minimal standard taught by all faculties. The advantage of such binding standard would be that respective expectation could be placed on prospective doctors, for example with regards to historical or ethical knowledge. Similarly, in view of the challenging teaching objectives, the understandable requirements for a higher allotment of hours and improved personnel resources in GTE institutes could gain in capacity through the communication of the knowledge or skills required for all medical students in Germany [15], [16]. On the other hand, some authors see the variance in education in GTE as a strength in an otherwise highly standardised and structured training curriculum [14]. An answer to the questions posed above is not only relevant for the established GTE institutions, but also for those new courses in human medicine currently developing which in parts are offered in co-operation with foreign institutions and with emphasis on other content aspects (e.g. combinations of ethics and medical law). 

### Limitations

The fact that about a quarter of the GTE institutions did not participate in the survey must be taken into account when interpreting the data. Furthermore, considering that in a number of cases GTE courses are taught either fully or partly by other institutions than GTE institutes, it is not possible to ascertain from the survey whether the answers were agreed upon in advance or whether distortions were caused by double entries. Due to anonymously collected data a plausibility test by way of a comparative assessment with other data sources (i.e. the institute’s Internet presence) was not possible as part of this investigation. Furthermore, in the authors’ estimation, the collection of qualitative data, for example, as a section of the partly structured research interviews, would be an expedient supplement, both in terms of method and content. The extent to which the triangulation of data – by way of various methods of data collection – leads to qualitative improvements in results for comparable future research projects requires verification due to dependency on existing resources. 

Some of the answers, particularly regarding information on the amount of GTE SWS and full-time equivalents, were not plausible; thus, in this case, it was not possible to evaluate part of the questionnaire. Finally, there are four faculties that conduct different curricula at the same time (i.e. regular and reformed/model curricula); hence, answers to parts of the questions, especially course content, methods and the corresponding evaluation, could only be carried out in a reduced form.

## 5. Conclusions

Content and structural preconditions of teaching GTE at medical faculties in Germany differ considerably. On the one hand, the variety of courses available may be understood as an advantage reflecting the broadness of the field, on the other hand, it seems necessary to consider whether, at least in reference to certain thematic areas, a standardisation of GTE teaching ought to be established. The advantage of defining the core content of GTE teaching is that all prospective doctors in Germany would have comparable fundamental knowledge. An obligatory GTE training content for doctors could also reinforce the position GTE as part of the medical undergraduate curriculum. 

## Authors

The authors Schildmann and Bruns contributed equally.

## Acknowledgements

The authors would like to thank those responsible for teaching GTE who were prepared to discuss the data collected following the survey and offer their estimation and interpretations of the results. The authors thank the expert reviewers for their constructive and important comments with respect to the revision.

## Competing interests

The authors declare that they have no competing interests.

## Figures and Tables

**Table 1:**
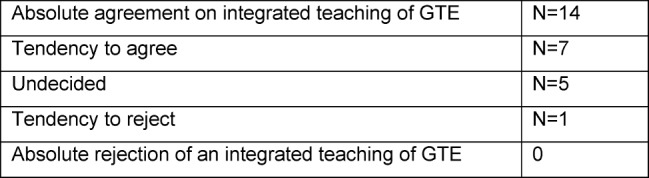
Agreement or rejection of an integrated mediation of historical, theoretical and ethical content as part of GTE courses

**Table 2:**
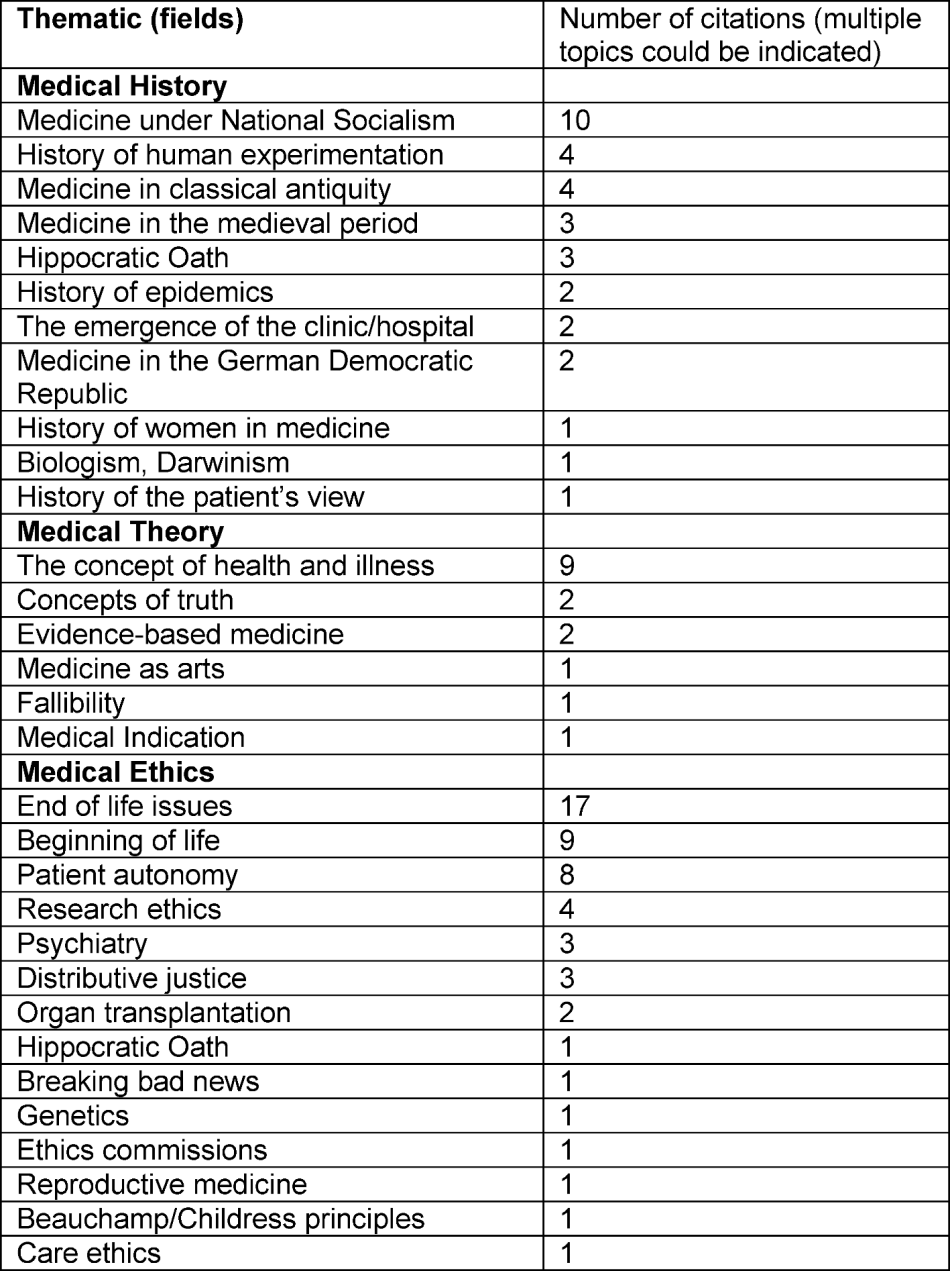
Subjects that should be taught as part of GTE according to those questioned
